# Poor Prognosis of Phosphatase of Regenerating Liver 3 Expression in Gastric Cancer: A Meta-Analysis

**DOI:** 10.1371/journal.pone.0076927

**Published:** 2013-10-18

**Authors:** Liren Hu, Haiqing Luo, Wenjuan Wang, Hongjiao Li, Taiping He

**Affiliations:** 1 Department of Epidemiology and Health Statistics, School of Public Health, Guangdong Medical College, Zhanjiang, Guangdong Province, China; 2 Center of Oncology, The Affiliated Hospital of Guangdong Medical College, Zhanjiang, Guangdong Province, China; 3 Department of Biochemistry and Molecular Biology, Guangdong Medical College, Zhanjiang, Guangdong Province, China; 4 Department of Nutrition and Food Hygiene, School of Public Health, Guangdong Medical College, Dongguan, Guangdong Province, China; The University of Kansas Medical Center, United States of America

## Abstract

**Background:**

Overexpression of phosphatase of regenerating liver 3 (PRL-3) has been implicated in gastric cancer (GC) metastasis. Epidemiological studies have evaluated the relationship between PRL-3 expression and prognosis in GC. However, results still remains controversial. In this study, a meta-analysis was performed to evaluate the association of PRL-3 expression with overall survival (OS) and clinicopathological characteristics.

**Methods:**

Literature databases were searched to identify eligible studies dated until April 2013. Summary hazard ratios (HRs) or odds ratios (ORs) with 95% confidence interval (95% CI) were calculated to estimate the association.

**Results:**

A total of 1380 GC patients from six studies were included in the meta-analysis. Overall, the combined HR estimate for OS in a random-effect model was 1.89 (95% CI = 1.38–2.60; *P*<0.001). Results showed that PRL-3 overexpression was significantly associated with OS, indicating that it may be a biomarker for poor prognosis of GC. Both subgroup and sensitivity analyses further identified the prognostic role of PRL-3 expression in GC patients. Moreover, PRL-3 overexpression was significantly associated with tumor stage (OR = 2.25; 95% CI = 1.63–3.12; *P*<0.001), depth of invasion (OR = 2.03; 95% CI = 1.38–2.98; *P*<0.001), vascular invasion (OR = 2.52; 95% CI = 1.79–3.56; *P*<0.001), lymphatic invasion (OR = 3.74; 95% CI = 2.49–5.63; *P*<0.001), and lymph node metastasis (OR = 4.56; 95% CI = 2.37–8.76; *P*<0.001). However, when age, sex, tumor size, and tumor differentiation were considered, no obvious association was observed.

**Conclusions:**

This meta-analysis reveals significant association of PRL-3 overexpression with OS and some clinicopathological features in GC. PRL-3 may be a predicative factor of poor prognosis and aggressive tumor behavior in GC patients.

## Introduction

The incidence and mortality of gastric cancer (GC) have dramatically decreased over the past few decades in many regions. However, this disease remains the second most common malignancy worldwide, with an estimated 989 600 new cases in 2008 [1], [2]. Despite advances in understanding the pathogenesis, early diagnosis, and new treatment approaches of GC, the results are still unsatisfactory [3]. Therefore, finding molecular markers that can predict the potential of tumor recurrence in GC patients and their prognosis is extremely important in establishing appropriate individualized therapy.

Considerable effort has been exerted to identify prognostic biomarkers in GC patients. Although some genes (e.g., CD133, Snail, p53, and STAT3) have been investigated in recent studies [4]–[7], a marker that can predict the survival of GC patients remains to be identified.

Protein tyrosine phosphatases (PTPs) are key regulatory enzymes in signal transduction pathways that are implicated in the tumorigenesis and metastasis of human cancers [8]. Phosphatase of regenerating liver (PRL) family is a PTP superfamily comprising three members, namely, PRL-1, PRL-2, and PRL-3 [9]. PRL-3 (also known as PTP4A3), is an important metastasis gene first identified in colorectal cancer in 2001 [10]. PRL-3 has been observed to be consistently overexpressed in all liver metastases derived from primary colorectal cancer compared with corresponding normal colorectal epithelium, adenomas, and primary tumors. Since then, many studies have suggested that PRL-3 expression is associated with metastasis of multiple tumor types by promoting the migration and invasion of tumor cells [11]–[13]. Therefore, the elevated expression of PRL-3 can be a significant biomarker for predicting poor survival in GC [14], [15].

Several studies have attempted to determine whether PRL-3 expression may be a prognostic factor for survival in GC patients. However, the results of these studies are controversial or inconclusive because of their limited sample size or genuine heterogeneity. Accordingly, this study aimed to review all available studies that investigated the relationship between PRL-3 overexpression and its clinical outcome in GC patients. A meta-analysis was conducted to more precisely estimate the prognostic significance of PRL-3 expression.

## Materials and Methods

### Search Strategy

The PubMed, ISI Web of Science, and Embase databases were searched to identify studies that assessed PRL-3 as a prognostic factor for survival in GC patients. The search ended in April 21, 2013, and no lower date limit was used. The search terms were “PRL-3,” “PRL3,” “PTP4A3,” “phosphatase of regenerating liver 3”; or “protein tyrosine phosphatase type IVA member 3” and “gastric tumors,” “gastric cancer,” “gastric carcinoma,” “gastric neoplasms,” “stomach cancer,” “stomachic cancer,” “stomachal cancer”; or “GC” and “survival,” “prognostic”; or “prognosis.” No language restriction was imposed. All references cited in the included studies were also reviewed to identify additional published articles not indexed in the common database.

### Study Eligibility

The studies included in this meta-analysis are either retrospective or prospective cohort studies that evaluated the association between PRL-3 expression and overall survival (OS; i.e., date of surgery to date of death as a result of any cause). Studies considered ineligible for the meta-analysis were as follows: reviews, conference abstracts, editorials, or letters; and articles with insufficient published data in a full-text paper for determining an estimate of hazard ratio (HR) and 95% confidence interval (CI). In case of multiple publications from the same institution with identical or overlapping patient cohorts, only the largest study was included to avoid duplication of information.

### Data Extraction

Two authors (Hu L.R. and Luo H.Q.) independently extracted data from eligible studies, and disagreements were resolved through consensus to all items. Standardized abstraction sheets were used to record data from individual studies. Data retrieved from the articles included the following: first author, year of publication, country of origin, ethnicity, number of patients analyzed, follow-up months, analysis method, blinding of PRL-3 measurements, cut-off value, number of high/low PRL-3 expression, HR estimation, and quality scores. For each study, HR was estimated using an approach reported by Parmar et al [16]. The most accurate approach is to obtain the HR estimate and 95% CI directly from the paper, or calculating them using the parameters such as the O-E statistic and variance offered in the manuscript. Otherwise, the number of patients at risk in each group, the number of events and *P*-value of the log-rank statistic were retrieved to permit an approximate calculation of the HR estimate and its variance. If the study did not provide a HR but reported the data in the form of the survival curve, survival rates at certain specified times were extracted from them for the reconstruction of the HR estimate and its variance, with the assumption that the rate of patients censored was constant during the follow-up.

### Quality Assessment

Quality assessment for the studies in this meta-analysis was performed using the Newcastle Ottawa scale (NOS) recommended by the Cochrane Non-Randomized Studies Methods Working Group [17], [18]. Based on the NOS, studies were judged based on three broad perspectives: selection of study groups (one criterion), comparability of study groups (four criteria), and ascertainment of outcome of interest (three criteria). Given the variability in quality of observational studies found on our initial literature search, we considered studies as high quality if they met five scores or more of the NOS criteria.

### Statistical Analysis

STATA version 11.0 (STATA Corporation, College Station, TX, USA) was used for all statistical analyses. For the pooled analysis of the correlation between PRL-3 overexpression and clinicopathological features (age, sex, tumor size, tumor stage, tumor differentiation, the depth of invasion, vascular invasion, lymphatic invasion, and lymph node metastasis), odds ratios (ORs) with their corresponding 95% CI were combined to estimate the effect. The combined HR with 95% CI was used to calculate and assess the strength of the association of PRL-3 expression. An observed HR>1 indicated a poor prognosis for the group with PRL-3 overexpression and would be considered to be statistically significant if the 95% CI did not overlap 1.

Heterogeneity assumption was examined by the chi-squared test based on the *Q* statistic [19] and was considered statistically significant when *P*<0.10. Heterogeneity was quantified by the *I* ^2^ metric, which is independent of the number of studies used in the meta-analysis (*I* ^2^<25%, no heterogeneity; *I* ^2^ = 25%–50%, moderate heterogeneity; *I* ^2^>50%, extreme heterogeneity). The pooled HR estimation of each study was calculated using a random-effects model (DerSimonian and Laird method) when *P*<0.10; otherwise, a fixed-effect model was used (Mantel–Haenszel method) [20].

To validate the credibility of outcomes in this meta-analysis, sensitivity analysis was performed by sequential omission of each individual study using the “metaninf” STATA command. Potential publication bias was evaluated through Begg’s and Egger’s Asymmetry tests [21], as well as through visual inspection of funnel plots, in which the standard error was plotted against log (HR) to form a simple scatterplot. Statistical significance for the interpretation of the Egger’s test was defined as *P*<0.10.

## Results

### Study Characteristics

**Figure 1:**
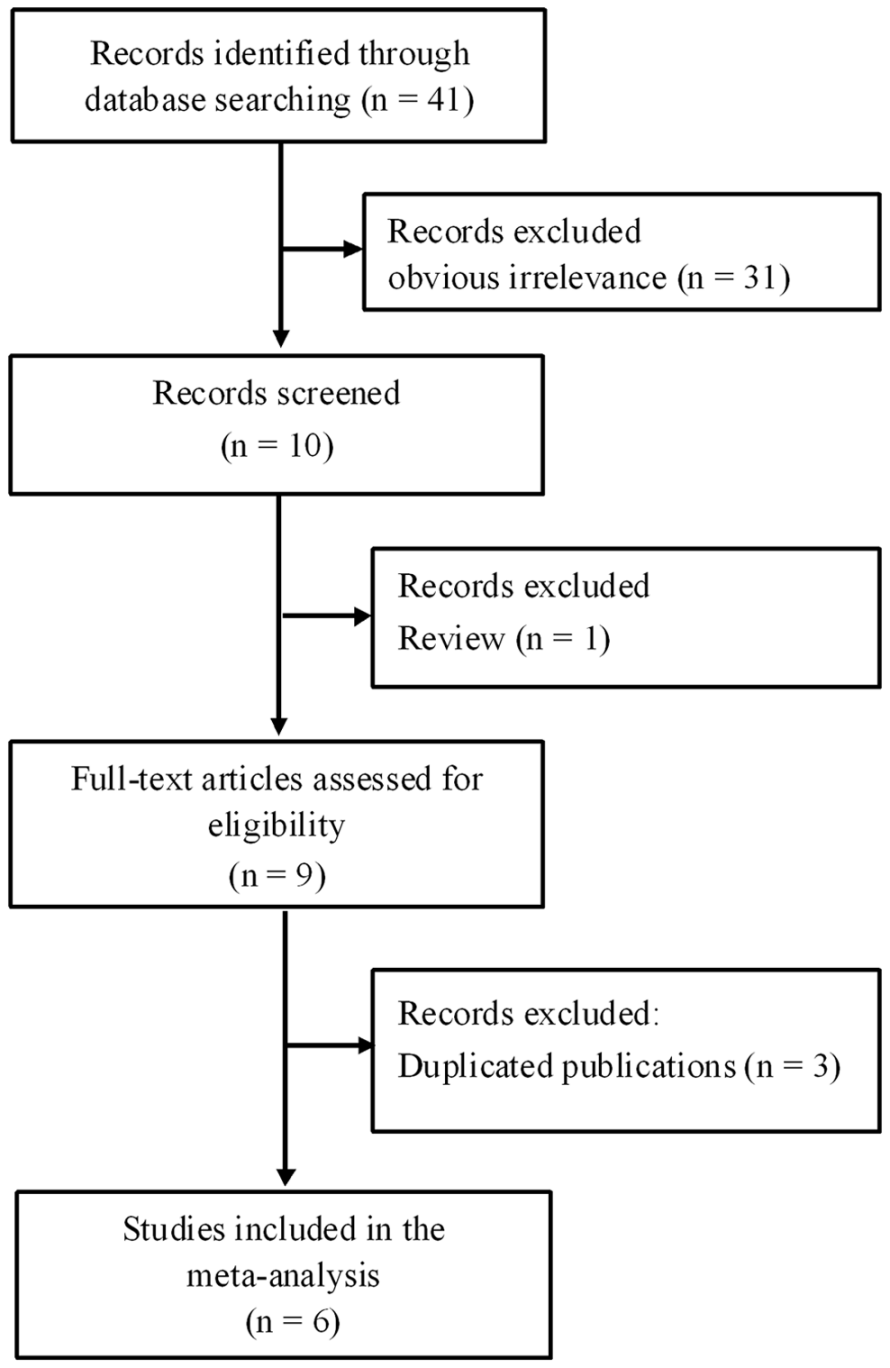
Flow diagram for study selection and specific reasons for exclusion in the meta-analysis.

Figure 1 illustrates the trial flow chart. The literature search identified a total of 41 potentially relevant articles. Upon further review, 31 were excluded after reading the title and abstract because of obvious lack of relevance. The following articles were also excluded: one review-type article [15], and three duplicated publications [22]–[24] overlapping populations with other eligible studies [25], [26]. After selection, six studies published in English language were finally enrolled for analysis of the prognostic value of PRL-3 expression in the meta-analysis [25]–[30].The main characteristics of these six included studies are summarized in Table 1. Among these studies, a total of 1380 GC patients ranging from 71 to 639 patients per study were reported to have been evaluated for the effect of PRL-3 expression on OS. The individual HRs of the included studies were calculated by one of the three methods reported in the “Data extraction” section. Three studies reported data from which the estimated HR can be directly retrieved [25], [26], [30]. For all other studies, HR had to be extrapolated from the survival curve [27]–[29]. Four out of six studies identified PRL-3 overexpression as an indicator of poor prognosis [25], [26], [28], [30], and all other studies showed no statistically significant effect of PRL-3 overexpression on survival period [27], [29]. According to the quality criteria, all studies were high quality. All included studies were retrospective cohort studies. Laboratory procedures for PRL-3 determination were reported in sufficient detail in all studies. For five reports, data on the association of PRL-3 and age, sex, and tumor stage can be obtained from published information; for four studies, information on the correlation of PRL-3 with tumor differentiation, depth of invasion, vascular invasion and lymph node metastasis can be extracted from the published articles. Five of the eligible studies clearly stated that PRL-3 determinations were blinded to outcomes [25]–[28], [30]. Information on the specified cutoff (5% or at least moderate staining) can be obtained in all enrolled studies.

**Table 1:** Main characteristic of 6 eligible studies enrolled in this meta-analysis.

Study (authors-year)	Patients source	Ethnicity	Recruitment time	No. of Patients	follow up (month)	PRL-3 expression (High/Low)	Analysis method	Blinding evaluation	Cutoff scores	Analysis of variance	HR estimation	Prognostic value	Quality score
Li ZR [25] (2007)	China	Asian	1994–2004	639	2–140	450/189	IHC	Yes	>5%	Multivariate	1.24(1.02–1.49)^a^	Poor	6
Miskad UA [27] (2007)	Japan	Asian	NR	94	0–52	34/60	ISH	Yes	>25%	Univariate	2.17(0.92–5.13)^b^	NS	5
Dai N [28] (2009)	China	Asian	1994.7–2000.12	293	0–122	127/166	IHC	Yes	>5%	Univariate	2.14(1.57–2.82)^b^	Poor	7
Ooki A [26] (2009)	Japan	Asian	1999–2003	173	0–60	95/78	IHC	Yes	Score≥2	Multivariate	2.74(1.53–4.90)^a^	Poor	6
Pryczynicz A [29] (2010)	Poland	Caucasian	NR	71	0–85	30/41	IHC	NR	>5%	Univariate	1.74(0.94–3.20)^b^	NS	5
Bilici A [30] (2012)	Turkey	Caucasian	2006.12–2009.10	110	3.5–58	79/31	IHC	Yes	Score≥2	Multivariate	2.25(1.32–3.84)^a^	Poor	6

*NR* data were not reported, *NS* not significant, *ISH* in situ hybridization, *IHC* immunohistochemistry,

a directly extracted from original data,

b extrapolated from survival curve.

### Correlation of PRL-3 with Clinicopathological Parameters

The associations of PRL-3 with clinicopathological characteristics were illustrated in Table 2 and Figure S1, S2, S3, S4, S5, S6, S7, S8, S9. Relationships existed between putative PRL-3 and biologically aggressive phenotypes such as tumor stage (pooled OR = 2.25; 95% CI = 1.63–3.12; *P*<0.001, fixed effect), depth of invasion (pooled OR = 2.03; 95% CI = 1.38–2.98; *P*<0.001, fixed effect), vascular invasion (pooled OR = 2.52; 95% CI = 1.79–3.56; *P*<0.001, fixed effect), lymphatic invasion (pooled OR = 3.74; 95% CI = 2.49–5.63; *P*<0.001, fixed effect), and lymph node metastasis (pooled OR = 4.56; 95% CI = 2.37–8.76; *P*<0.001, random effect). These findings suggested that PRL-3 overexpression was obviously associated with tumor stage, extent of invasion, and lymph node metastasis. However, no association existed between PRL-3 and other clinicopathological parameters such as age (pooled OR = 0.88; 95% CI = 0.55–1.38; *P* = 0.566, random effect), sex (pooled OR = 1.18; 95% CI = 0.86–1.62; *P* = 0.306, fixed effect), tumor size (pooled OR = 1.61; 95% CI = 0.76–3.42; *P* = 0.217, random effect), and tumor differentiation (pooled OR = 1.12; 95% CI = 0.81–1.55; *P* = 0.496, fixed effect).

**Table 2:** Meta-analysis of PRL-3 overexpression and clinicopathological features in gastric cancer patients.

Categories	Studies (patients)	OR (95% CI)	*I* ^2^ (%)	*P*_h_	*Z*	*P*
Age	5(741)	0.88(0.55–1.38)	50.0	0.092	0.57	0.566
Sex	5(741)	1.18(0.86–1.62)^F^	30.0	0.221	1.02	0.306
Tumor size	3(497)	1.61(0.76–3.42)	68.0	0.044	1.24	0.217
Tumor differentiation	4(647)	1.12(0.81–1.55)^F^	50.5	0.109	0.68	0.496
Tumor stage	5(741)	2.25(1.63–3.12)^F^	43.8	0.130	4.92	<0.001
Depth of invasion	4(631)	2.03(1.38–2.98)^F^	0.0	0.840	3.59	<0.001
Vascular invasion	4(670)	2.52(1.79–3.56)^F^	43.8	0.149	5.29	<0.001
Lymphatic invasion	3(560)	3.74(2.49–5.63)^F^	17.7	0.293	6.33	<0.001
Lymph node metastasis	4(670)	4.56(2.37–8.76)	67.9	0.025	4.56	<0.001

All pooled *OR* ***s*** were derived from random-effect model except for cells marked with (fixed^F^).

*P*_h_ *P*-value for heterogeneity based on ***Q*** test.

*P P*-value for statistical significance based on ***Z*** test.

### Correlation of PRL-3 with Survival

**Figure 2:**
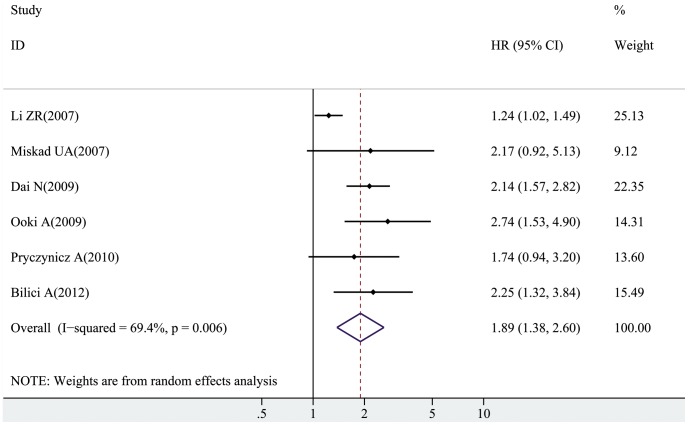
The forest plot for the overall association between PRL-3 overexpression and OS of GC patients. The contribution of each study to the meta-analysis (its weight) is represented by the *area* of a *box*, the *center* of which represents the size of the HR estimated from that study. The 95% CI for the HR (*extending lines*) from each study is also shown. The pooled HR is shown in the *middle* of a *diamond*, the *left* and *right* extremes of which represent the corresponding CI.

We also carried out a meta-analysis on the association of PRL-3 overexpression in GC patients with OS. The pooled HRs along with their 95% CI were presented in detail in Table 3. A poor prognosis was demonstrated in the overall HR estimate (pooled HR = 1.89; 95% CI = 1.38–2.60; *Z* = 3.95; *P*<0.001, random effect), although a significant degree of heterogeneity (*I* ^2^ = 69.4%, *P*_h_ = 0.006) was present. Further analysis was performed on data stratified by variance analysis to determine possible factors that may have influenced the results. Results showed that poor prognosis was found in GC patients with PRL-3 overexpression under multivariate analyses (pooled HR = 1.87; 95% CI = 1.08–3.23; *Z = *2.23; *P* = 0.026, random effect) and univariate analyses (pooled HR = 2.07; 95% CI = 1.61–2.66; *Z* = 5.64; *P*<0.001, random effect). When subgrouped by ethnicity, unfavorable survival results were observed in patients from Asian populations (pooled HR = 1.88; 95% CI = 1.23–2.88; *Z = *2.92; *P* = 0.004, random effect) and Caucasian populations (pooled HR = 2.01; 95% CI = 1.35–3.01; *Z = *3.41; *P*<0.001, random effect). After exclusion of the study without blinded evaluation [29], the pooled HR was 1.94 (95% CI = 1.34–2.79; *Z* = 3.54; *P*<0.001, random effect); after omission of the study [27] using in situ hybridization not immunohistochemistry analysis yielded a pooled result of 1.87 (95% CI = 1.33–2.65; *Z* = 3.57; *P*<0.001, random effect). Furthermore, a prognostic effect on survival was also observed in the three largest studies [25], [26], [28]. Results did not change when cutoff values were considered. The forest plot for the overall association of PRL-3 overexpression with OS in GC patients was shown in Figure 2.

**Table 3:** Meta-analysis of PRL-3 overexpression and prognosis in gastric cancer patients.

Categories	Studies(patients)	HR (95% CI)	*I* ^2^ (%)	*P*_h_	*Z*	*P*
Overallsurvival	6(1380)	1.89(1.38,2.60)	69.4	0.006	3.95	<0.001
Multivariateanalyses	3(922)	1.87(1.08,3.23)	79.6	0.002	2.23	0.026
Univariateanalyses	3(458)	2.07(1.61,2.66)	60.9	0.008	5.64	<0.001
Asian	4(1199)	1.88(1.23,2.88)	79.2	0.002	2.92	0.004
Caucasian	2(181)	2.01(1.35,3.01)	67.0	0.009	3.41	<0.001
IHCanalysis	5(1286)	1.87(1.33,2.65)	74.7	0.003	3.57	<0.001
Statedblinding	5(1309)	1.94(1.34,2.79)	75.4	0.003	3.54	<0.001
Largeststudies	3(1105)	1.84(1.14–2.99)	85.5	0.010	2.49	0.013
Cutoffvalue >5%	3(1003)	1.63(1.08–2.47)	79.7	0.007	2.31	0.021

All pooled *HR* ***s*** were derived from random-effect model.

*P*_h_ *P*-value for heterogeneity based on ***Q*** test.

*P P*-value for statistical significance based on ***Z*** test.

### Sensitivity Analysis and Publication Bias

**Figure 3:**
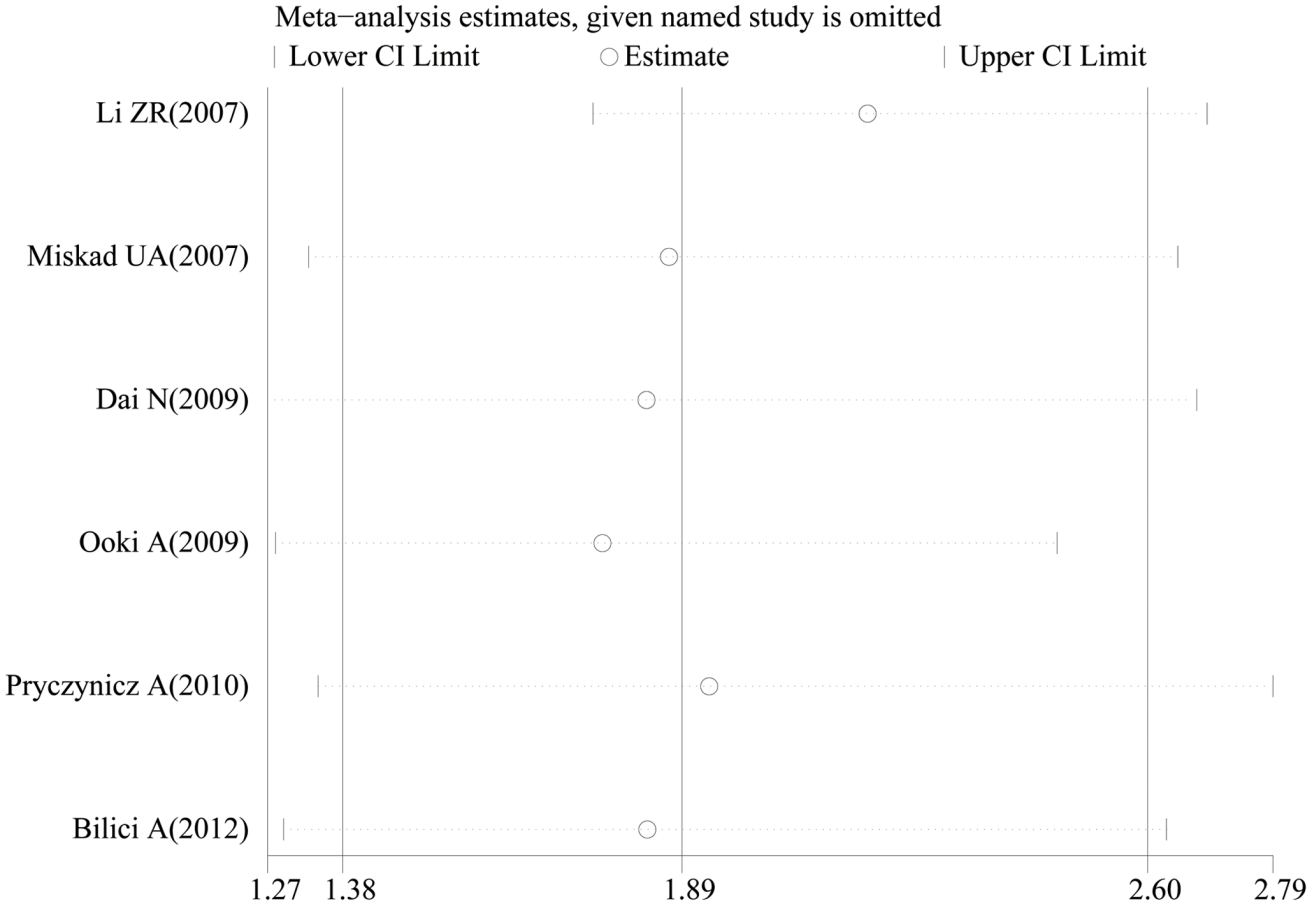
Effect of individual studies on the pooled HR for PRL-3 overexpression and OS of GC patients. The *vertical axis* at 1.89 indicates the overall HR, and the *two vertical axes* at 1.38 and 2.60 indicate the 95% CI. Every *hollow round* indicates the pooled HR when the left study was omitted in a meta-analysis with a random model. The *two ends* of every *broken line* represent the respective 95% CI.

In the sensitivity analysis, the influence of each study on the pooled HR was examined by repeating the meta-analysis while omitting each study one at a time. Figure 3 demonstrated that no point estimate of the omitted individual study lied outside the 95% CI of the combined analysis on the summary OS. These analyses suggested that no individual study dominated the results in the meta-analysis, which validated the credibility of outcomes.

Publication bias was analyzed in the included literature involving the overall HR estimation of OS. No obvious publication bias was detected in either Begg’s test (*Z* = 0.00; *P* = 1.000) or the Egger’s tests (*t* = 2.10; *P* = 0.103; 95% CI = −0.78–5.64). The shapes of the funnel plots also showed that the included studies did not have apparent asymmetry, thereby indicating that our results were statistically robust.

## Discussion and Conclusion

Since PRL-3 protein was firstly determined to play a key role in tumor metastatic process, the biological functions of this protein have been extensively studied by in vitro experiments and in vivo analyses. Moreover, PRL-3 expression is reportedly a potential prognostic factor in different types of cancer, including colorectal [31], ovarian [32], hepatocellular [33], nasopharyngeal [34], and breast [35] cancers.

The relationship between GC prognosis and PRL-3 expression has also attracted considerable attention. Li et al. [25] reported that individuals with highly expressed PRL-3 have a significantly shorter survival than individuals with no or low expression genotype. However, some researchers such as Miskad et al. [27] and Pryczynicz et al. [29] failed to demonstrate any relationship between PRL-3 overexpression and survival in GC patients. These controversies in the predictive significance of the PRL-3 phenotype in GC warrant a quantitative meta-analysis of the study outcomes.

To our knowledge, the present meta-analysis is the first study to systematically elucidate the association of PRL-3 expression with OS and clinicopathological characteristics of GC. Results showed that PRL-3 overexpression was significantly associated with OS, indicating that PRL-3 may be a marker for poor prognosis of GC. All subgroup analyses and sensitivity analyses identified the prognostic role of PRL-3 overexpression in GC patients. Notably, when the analysis was restricted to multivariate analyses, a statistically significant unfavorable effect of PRL-3 overexpression on OS was observed. Thus, PRL-3 expression may be an independent factor for OS. Furthermore, significant correlations were observed between PRL-3 overexpression and clinicopathological features, including tumor stage, depth of invasion, vascular invasion, lymphatic invasion, and lymph node metastasis, which revealed that PRL-3 may facilitate invasion and metastasis. Begg’s test, Egger’s test, and funnel plot revealed no publication bias in our analysis. Regarding quality assessment, all included studies in the meta-analysis were high quality (quality scores ≥5). Thus, the results are encouraging and may provide further basis for the development of new markers for GC prognosis and of PRL-3 inhibitors for individualized therapy.

However, the results should be interpreted very cautiously. In this review, the test for heterogeneity of the included studies was significant. Although we used subgroup analyses by variance analysis, ethnicity, PRL-3 measurements, blinded evaluation, and the number of patients during pooling data, all stratified analyses did not identify the source of heterogeneity. Moreover, sensitivity analysis did not help clarify the source of heterogeneity in this study. Therefore, multidimensional network meta-analysis models for published survival curves can be established to explain systematic heterogeneity across studies and to reduce inconsistencies [36].

Although the present meta-analysis had some advantages over other individual studies, a few limitations were also inherent. First was our inability to explore the potential effect of confounding factors, such as tumor location and different treatment regimens, because of insufficient information in the included studies. Second, the studies included in the meta-analysis were from different sources of PRL-3 antibody and dilutions of the antibodies, indicating a possibility that antibody factors can confound the results. Third, differences in the cutoff definition of PRL-3 overexpression and the experimental processes may partly influence the significance of the clinicopathological outcome in survival analyses and partially account for the inter-study heterogeneity. The fourth and last limitation is related to the approach of the HRs and 95% CI estimations. In the meta-analysis, HRs and 95% CI were directly extracted from original data in three included studies. For all other studies, HR had to be extrapolated from the survival curve. Thus, the estimated HR may be less reliable than when directly obtained from published statistics [37]. Meanwhile, we compared our estimated HRs and their statistical significance with the results reported in papers and did not identify any major deviation.

We hereby make the following recommendations to future studies: 1) a large series of consecutive patients from a single cohort, 2) sufficient long-term follow-up, 3) usage of monoclonal instead of polyclonal antibody directed against PRL-3 for immunostaining, 4) uniform standard for assessment overexpression, 5) complete description of the clinical characteristics of a study population, 6) presentation of results as survival curves and as a multivariate Cox proportional hazard model, and 7) complete description of survival events to time. Moreover, further studies should include more homogeneous populations and be prospective.

In summary, this meta-analysis revealed that PRL-3 overexpression was significantly associated with poor OS and clinicopathological features in GC. PRL-3 expression may be a predicative factor of poor prognosis and aggressive tumor behavior in GC patients.

## Supporting Information

Figure S1 **The forest plot for the overall association between PRL-3 overexpression and age in GC patients.** The contribution of each study to the meta-analysis (its weight) is represented by the *area* of a *box*, the *center* of which represents the size of the OR estimated from that study. The 95% CI for the OR (*extending lines*) from each study is also shown. The pooled OR is shown in the *middle* of a *diamond*, the *left* and *right* extremes of which represent the corresponding CI.
(TIF)

Figure S2 **The forest plot for the overall association between PRL-3 overexpression and sex of GC patients.** The contribution of each study to the meta-analysis (its weight) is represented by the *area* of a *box*, the *center* of which represents the size of the OR estimated from that study. The 95% CI for the OR (*extending lines*) from each study is also shown. The pooled OR is shown in the *middle* of a *diamond*, the *left* and *right* extremes of which represent the corresponding CI.
(TIF)

Figure S3 **The forest plot for the overall association between PRL-3 overexpression and tumor size of GC patients.** The contribution of each study to the meta-analysis (its weight) is represented by the *area* of a *box*, the *center* of which represents the size of the OR estimated from that study. The 95% CI for the OR (*extending lines*) from each study is also shown. The pooled OR is shown in the *middle* of a *diamond*, the *left* and *right* extremes of which represent the corresponding CI.
(TIF)

Figure S4 **The forest plot for the overall association between PRL-3 overexpression and tumor differentiation of GC patients.** The contribution of each study to the meta-analysis (its weight) is represented by the *area* of a *box*, the *center* of which represents the size of the OR estimated from that study. The 95% CI for the OR (*extending lines*) from each study is also shown. The pooled OR is shown in the *middle* of a *diamond*, the *left* and *right* extremes of which represent the corresponding CI.
(TIF)

Figure S5 **The forest plot for the overall association between PRL-3 overexpression and tumor stage of GC patients.** The contribution of each study to the meta-analysis (its weight) is represented by the *area* of a *box*, the *center* of which represents the size of the OR estimated from that study. The 95% CI for the OR (*extending lines*) from each study is also shown. The pooled OR is shown in the *middle* of a *diamond*, the *left* and *right* extremes of which represent the corresponding CI.
(TIF)

Figure S6 **The forest plot for the overall association between PRL-3 overexpression and depth of invasion of GC patients.** The contribution of each study to the meta-analysis (its weight) is represented by the *area* of a *box*, the *center* of which represents the size of the OR estimated from that study. The 95% CI for the OR (*extending lines*) from each study is also shown. The pooled OR is shown in the *middle* of a *diamond*, the *left* and *right* extremes of which represent the corresponding CI.
(TIF)

Figure S7 **The forest plot for the overall association between PRL-3 overexpression and vascular invasion of GC patients.** The contribution of each study to the meta-analysis (its weight) is represented by the *area* of a *box*, the *center* of which represents the size of the OR estimated from that study. The 95% CI for the OR (*extending lines*) from each study is also shown. The pooled OR is shown in the *middle* of a *diamond*, the *left* and *right* extremes of which represent the corresponding CI.
(TIF)

Figure S8 **The forest plot for the overall association between PRL-3 overexpression and lymphatic invasion of GC patients.** The contribution of each study to the meta-analysis (its weight) is represented by the *area* of a *box*, the *center* of which represents the size of the OR estimated from that study. The 95% CI for the OR (*extending lines*) from each study is also shown. The pooled OR is shown in the *middle* of a *diamond*, the *left* and *right* extremes of which represent the corresponding CI.
(TIF)

Figure S9 **The forest plot for the overall association between PRL-3 overexpression and lymph node metastasis of GC patients.** The contribution of each study to the meta-analysis (its weight) is represented by the *area* of a *box*, the *center* of which represents the size of the OR estimated from that study. The 95% CI for the OR (*extending lines*) from each study is also shown. The pooled OR is shown in the *middle* of a *diamond*, the *left* and *right* extremes of which represent the corresponding CI.
(TIF)

Checklist S1 **Prisma checklist.**
(DOC)
