# Supplementary figures and images for: Poor Prognosis of Phosphatase of Regenerating Liver 3 Expression in Gastric Cancer: A Meta-Analysis
Source: PLoS One. 2013 Oct 18;8(10):e76927. doi: 10.1371/journal.pone.0076927 (PMC3799911; doi:10.1371/journal.pone.0076927)

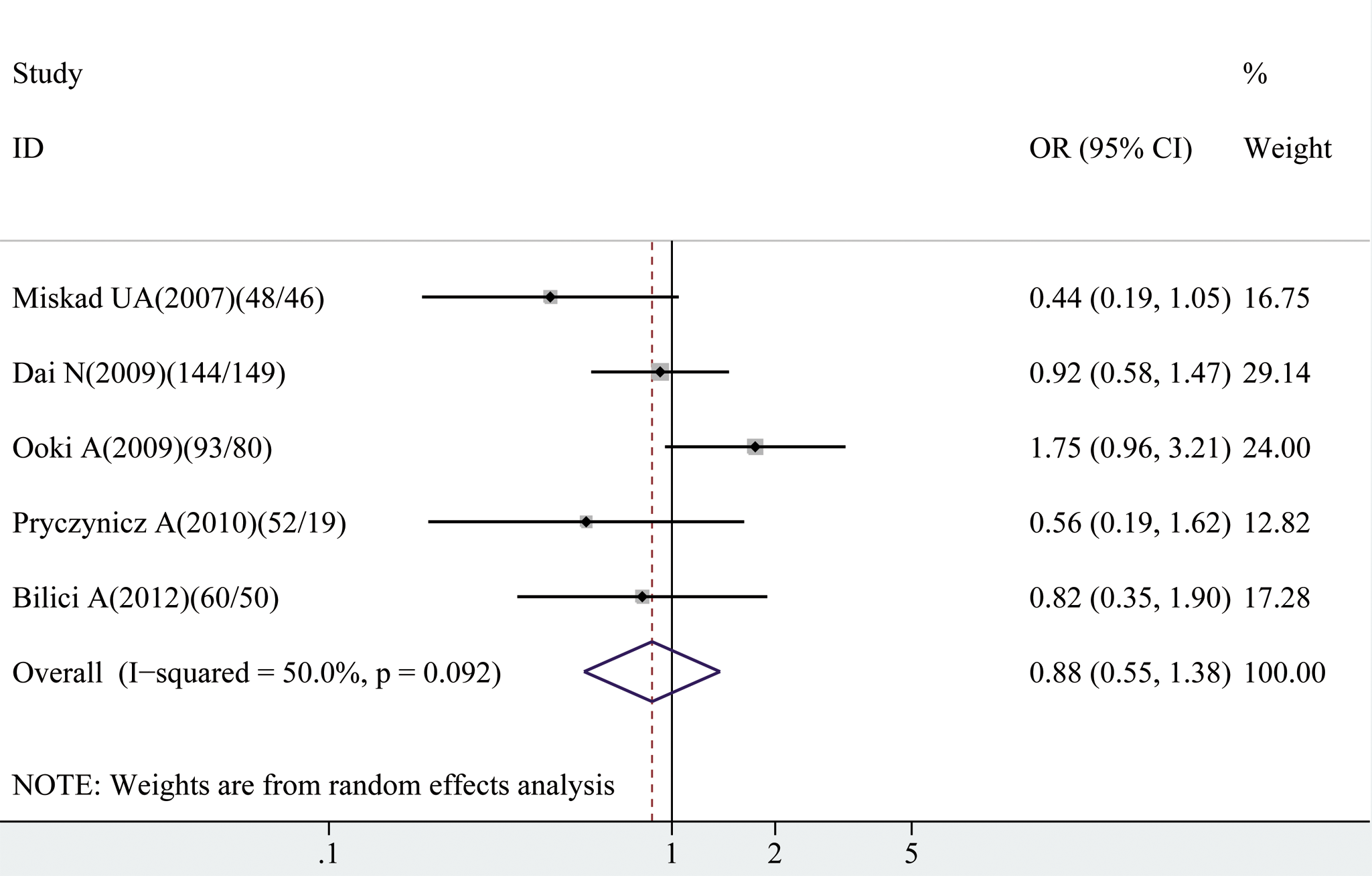

Supplement: Figure S1 — The forest plot for the overall association between PRL-3 overexpression and age in GC patients. The contribution of each study to the meta-analysis (its weight) is represented by the area of a box, the center of which represents the size of the OR estimated from that study. The 95% CI for the OR (extending lines) from each study is also shown. The pooled OR is shown in the middle of a diamond, the left and right extremes of which represent the corresponding CI. (TIF) [file pone.0076927.s001.tif]

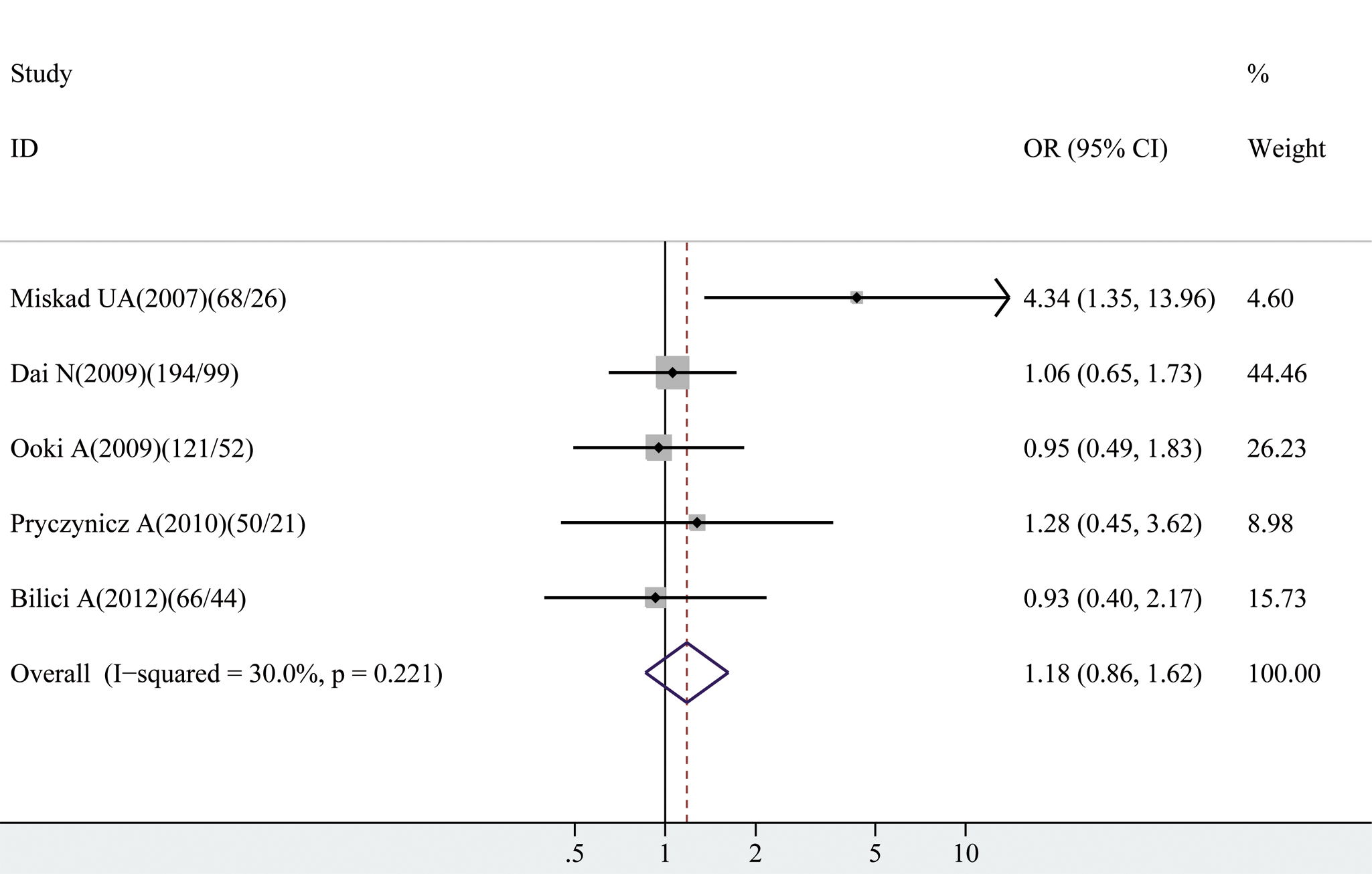

Supplement: Figure S2 — The forest plot for the overall association between PRL-3 overexpression and sex of GC patients. The contribution of each study to the meta-analysis (its weight) is represented by the area of a box, the center of which represents the size of the OR estimated from that study. The 95% CI for the OR (extending lines) from each study is also shown. The pooled OR is shown in the middle of a diamond, the left and right extremes of which represent the corresponding CI. (TIF) [file pone.0076927.s002.tif]

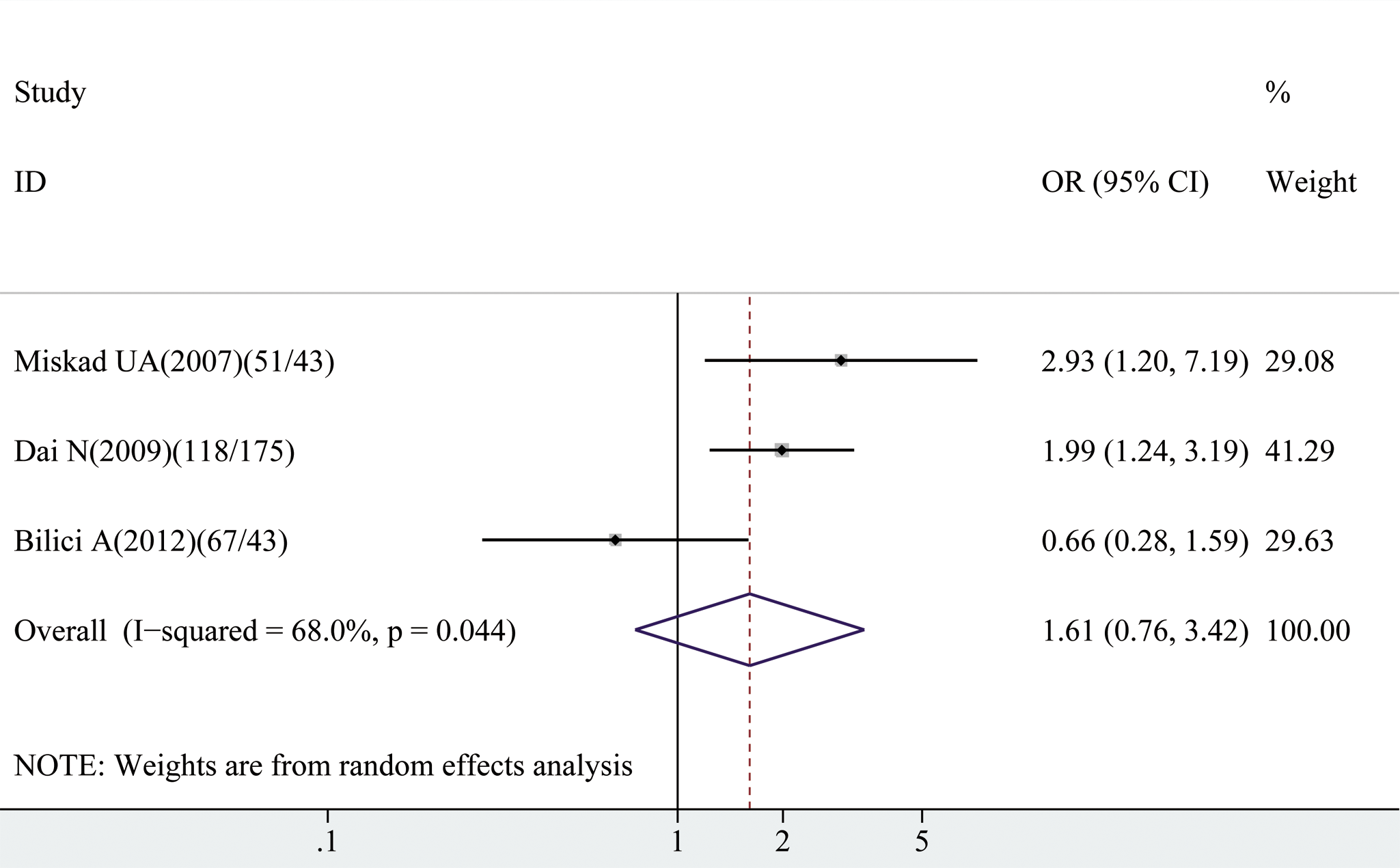

Supplement: Figure S3 — The forest plot for the overall association between PRL-3 overexpression and tumor size of GC patients. The contribution of each study to the meta-analysis (its weight) is represented by the area of a box, the center of which represents the size of the OR estimated from that study. The 95% CI for the OR (extending lines) from each study is also shown. The pooled OR is shown in the middle of a diamond, the left and right extremes of which represent the corresponding CI. (TIF) [file pone.0076927.s003.tif]

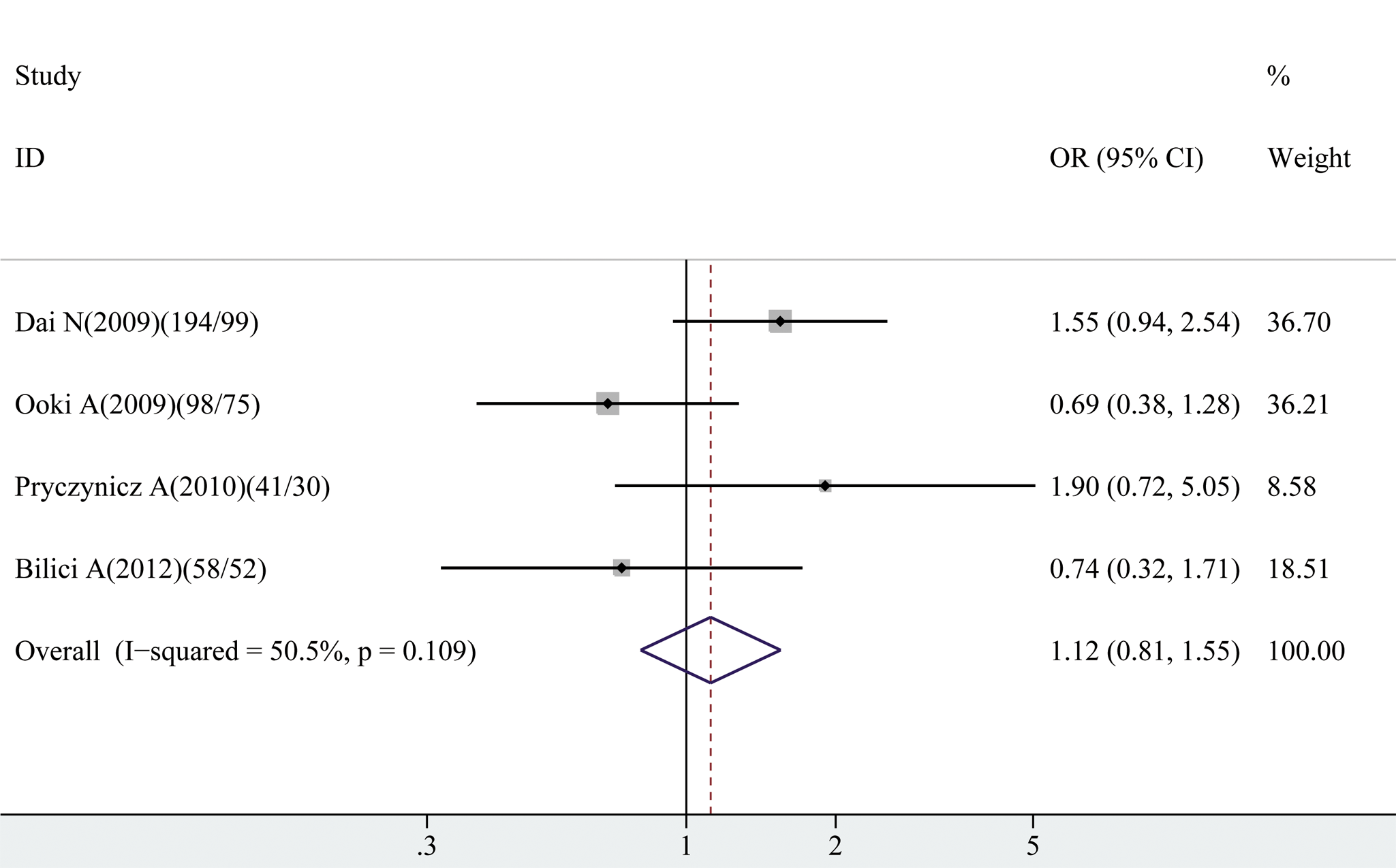

Supplement: Figure S4 — The forest plot for the overall association between PRL-3 overexpression and tumor differentiation of GC patients. The contribution of each study to the meta-analysis (its weight) is represented by the area of a box, the center of which represents the size of the OR estimated from that study. The 95% CI for the OR (extending lines) from each study is also shown. The pooled OR is shown in the middle of a diamond, the left and right extremes of which represent the corresponding CI. (TIF) [file pone.0076927.s004.tif]

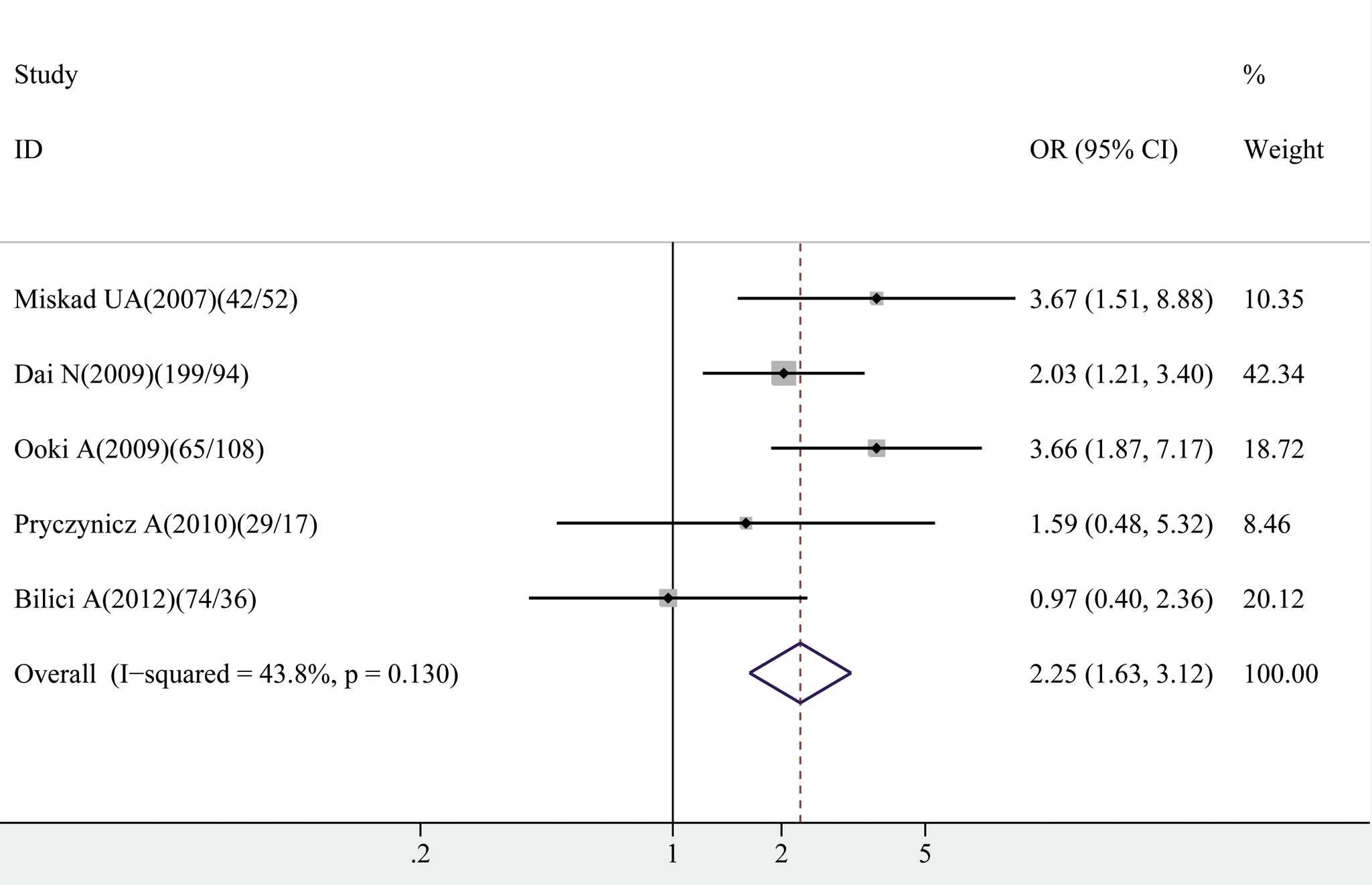

Supplement: Figure S5 — The forest plot for the overall association between PRL-3 overexpression and tumor stage of GC patients. The contribution of each study to the meta-analysis (its weight) is represented by the area of a box, the center of which represents the size of the OR estimated from that study. The 95% CI for the OR (extending lines) from each study is also shown. The pooled OR is shown in the middle of a diamond, the left and right extremes of which represent the corresponding CI. (TIF) [file pone.0076927.s005.tif]

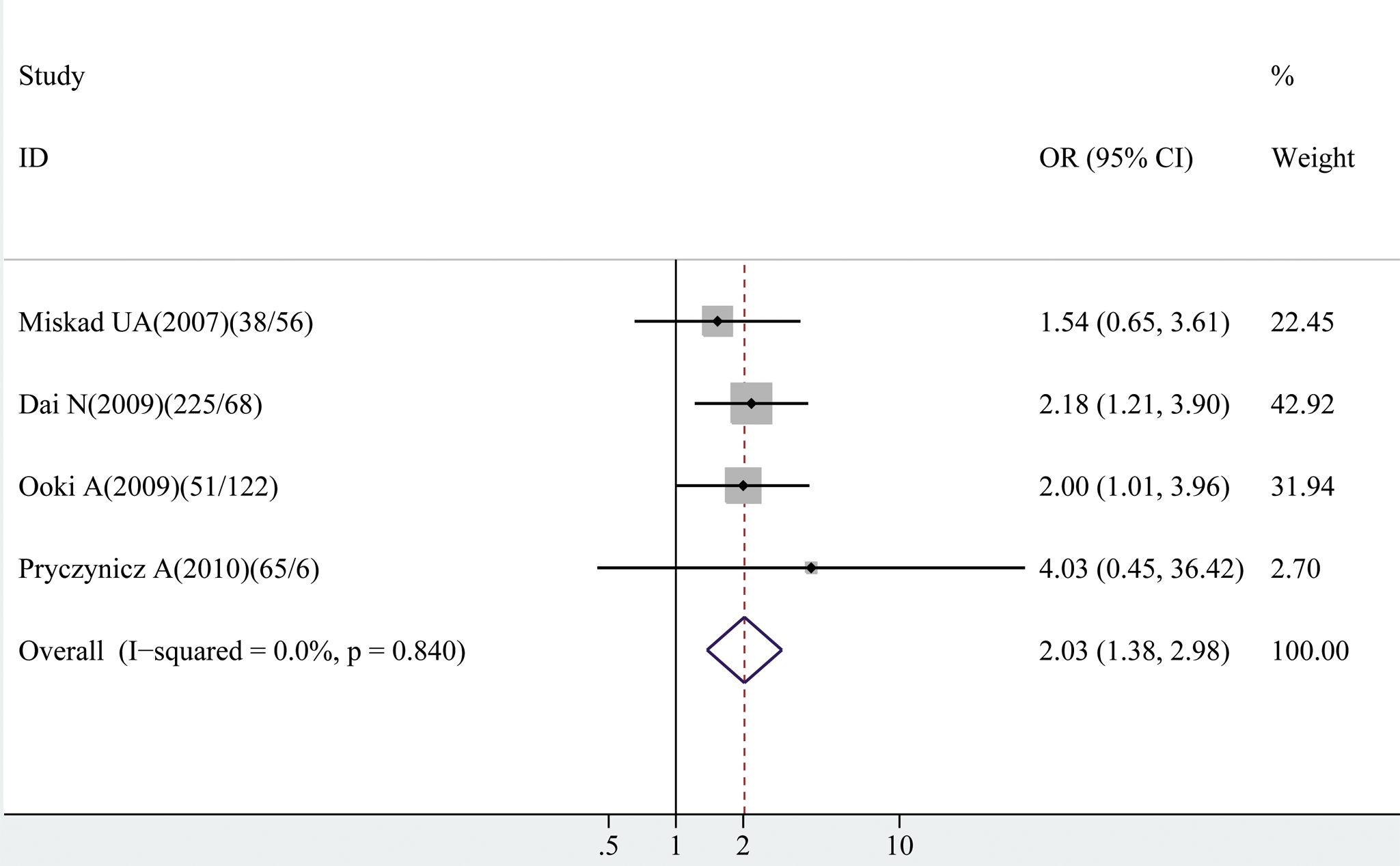

Supplement: Figure S6 — The forest plot for the overall association between PRL-3 overexpression and depth of invasion of GC patients. The contribution of each study to the meta-analysis (its weight) is represented by the area of a box, the center of which represents the size of the OR estimated from that study. The 95% CI for the OR (extending lines) from each study is also shown. The pooled OR is shown in the middle of a diamond, the left and right extremes of which represent the corresponding CI. (TIF) [file pone.0076927.s006.tif]

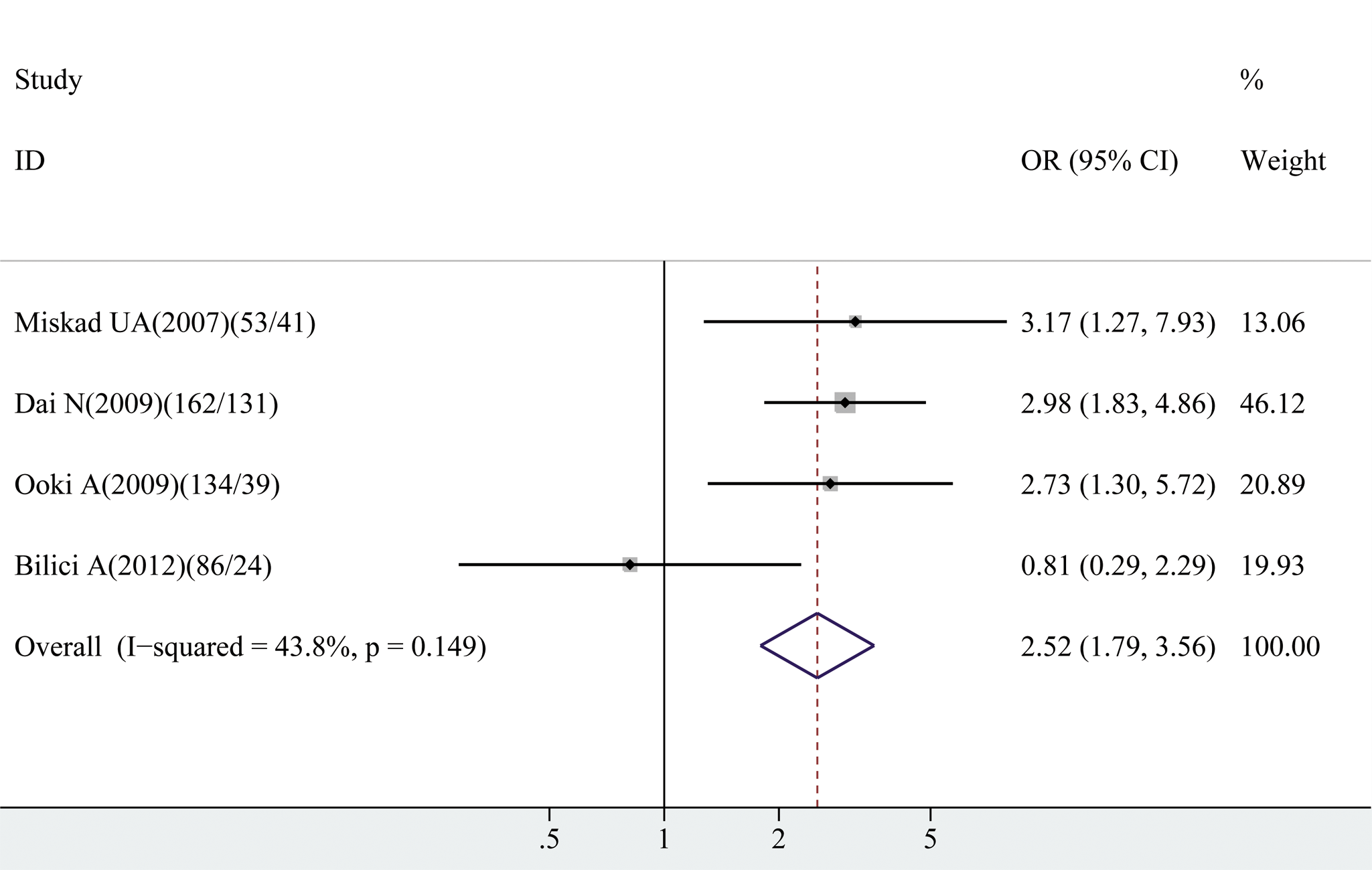

Supplement: Figure S7 — The forest plot for the overall association between PRL-3 overexpression and vascular invasion of GC patients. The contribution of each study to the meta-analysis (its weight) is represented by the area of a box, the center of which represents the size of the OR estimated from that study. The 95% CI for the OR (extending lines) from each study is also shown. The pooled OR is shown in the middle of a diamond, the left and right extremes of which represent the corresponding CI. (TIF) [file pone.0076927.s007.tif]

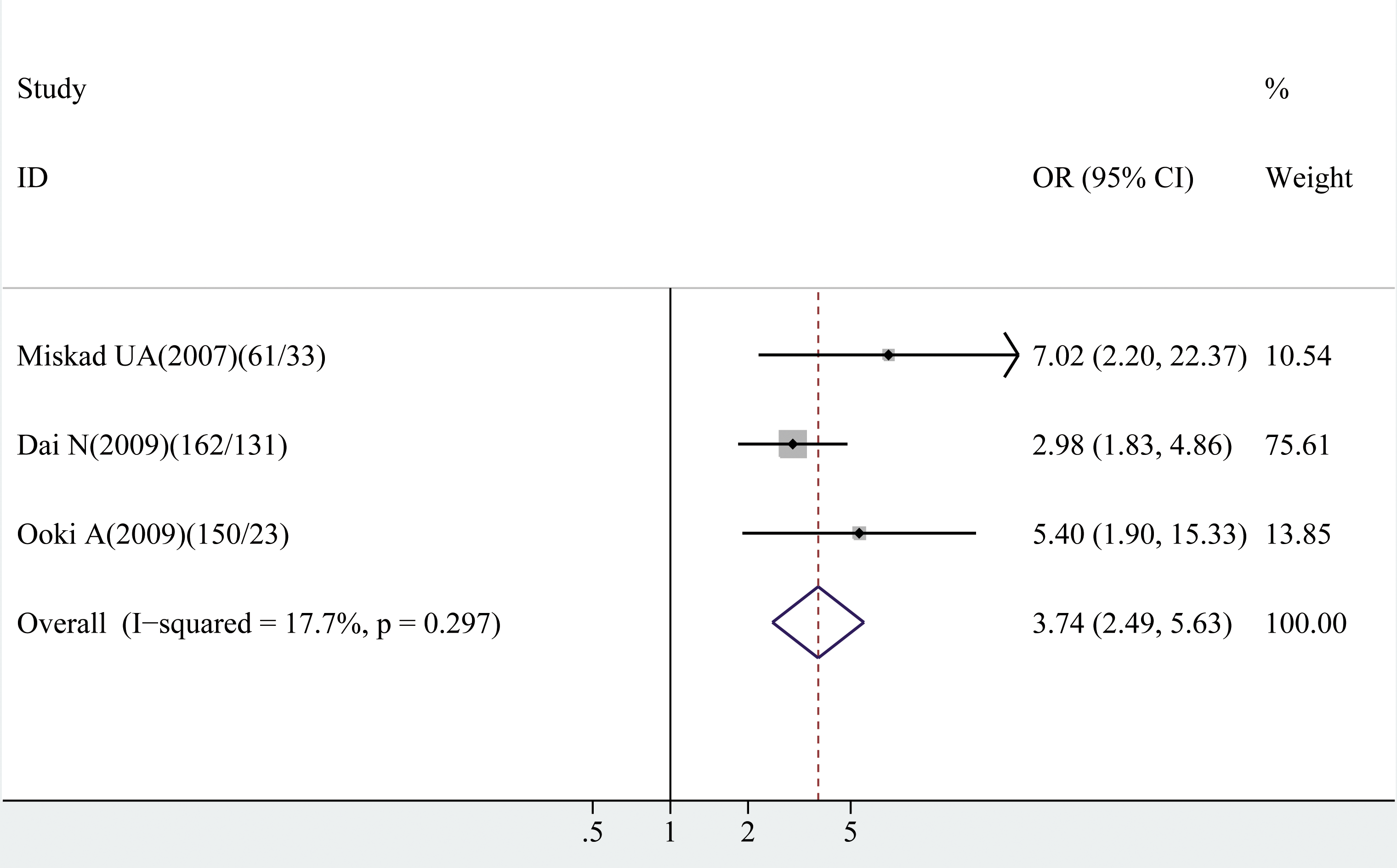

Supplement: Figure S8 — The forest plot for the overall association between PRL-3 overexpression and lymphatic invasion of GC patients. The contribution of each study to the meta-analysis (its weight) is represented by the area of a box, the center of which represents the size of the OR estimated from that study. The 95% CI for the OR (extending lines) from each study is also shown. The pooled OR is shown in the middle of a diamond, the left and right extremes of which represent the corresponding CI. (TIF) [file pone.0076927.s008.tif]

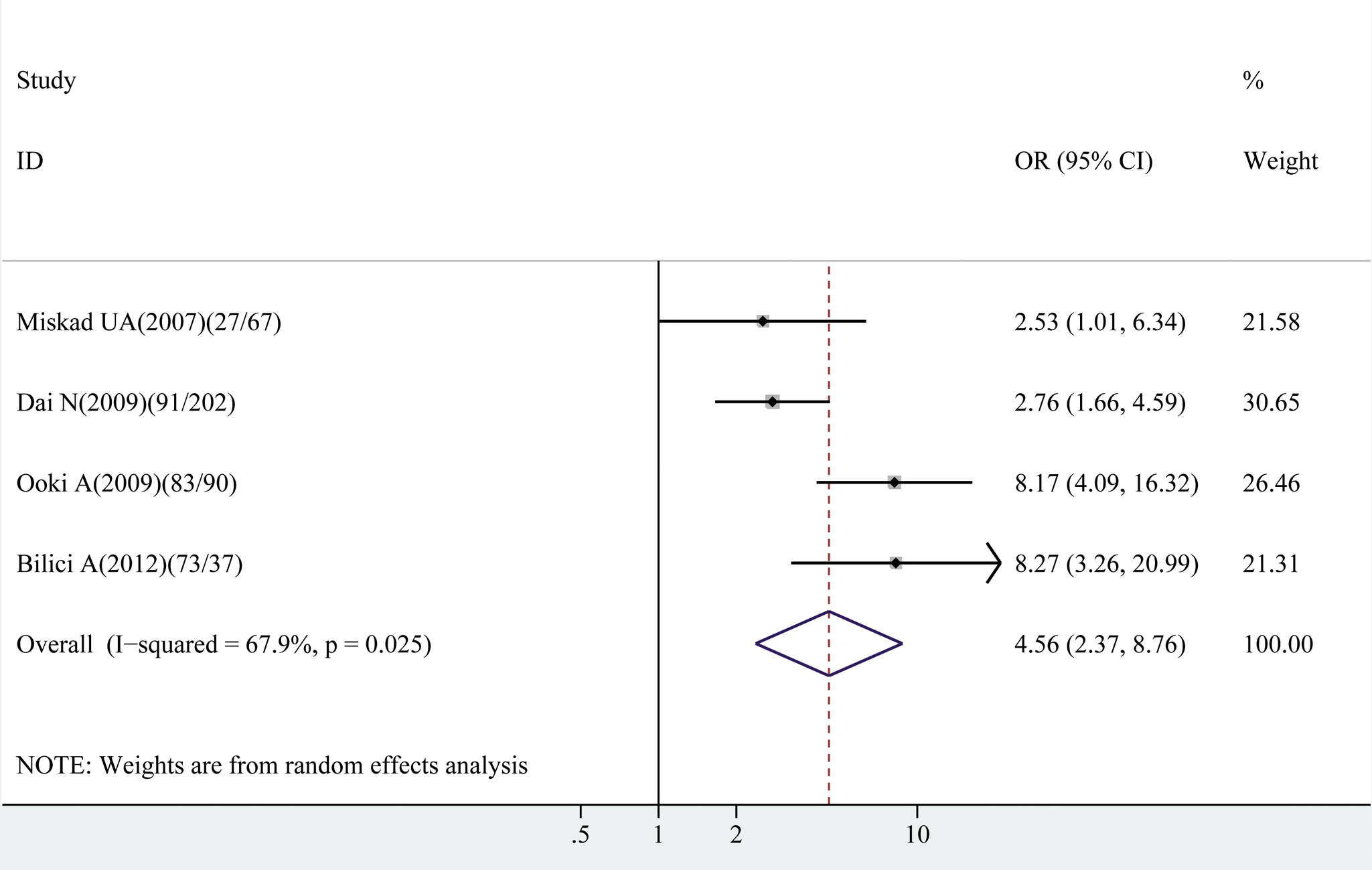

Supplement: Figure S9 — The forest plot for the overall association between PRL-3 overexpression and lymph node metastasis of GC patients. The contribution of each study to the meta-analysis (its weight) is represented by the area of a box, the center of which represents the size of the OR estimated from that study. The 95% CI for the OR (extending lines) from each study is also shown. The pooled OR is shown in the middle of a diamond, the left and right extremes of which represent the corresponding CI. (TIF) [file pone.0076927.s009.tif]
